# Book Reviews

**Published:** 1799-04

**Authors:** 


### CHEMISTRY AND NATURAL PHILOSOPHY.

*Cours elementaire de chymie theorique et pratique, &c.*—"*An elementary View of the Theory and Practice of Chemistry, according to the new Nomenclature; together with the most useful and entertaining Experiments made in this Science.*" By Cit. Alyon, Officer of Health in the Military Hospital of Val-de-grace. 2 volumes 8vo. 1798—99. Paris, Published by the Author.

The contents of this excellent treatise fully correspond to the professions of its title, and it is written in a plain and perspicuous style. Although it is chiefly designed for the use of the tyro, it well deserves to be recommended also to the attentive perusal of the practical chemist, as it abounds not only in new and original experiments, but likewise in chemical preparations, the various processes of which had not hitherto been published. Among other medicinal articles, the author gives directions for preparing an oxygenated ointment *(pomade oxygenée)* by the successful application of which he has obtained considerable celebrity among the Parisians, in the cure of lues venerea.

*Elements of Chemistry*: by Joseph Francis Jacquin, Professor of Chemistry and Botany, at Vienna; Fellow of the Linnæan Society of London, and Member of several Academies of Science, Agriculture, &c. *Translated from the German.* 8vo. 428 pp. with Preface, Table of Contents, &c. with a Plate representing Woulfe's Apparatus for Compound Distillation. *London*; Murray and Highley. *Edinburgh*; W. Mudie. 1799. 7s. 6d.

From the advertisement prefixed to this volume, we learn that it is translated by a Mr. Henry Stutzer, who, at the same time informs us, that these Elements were originally written with a view to convey to the young student a knowledge of the most important truths in chemistry. We agree with the translator, that this work is written by Mr. Jacquin, 'with accuracy and simplicity': we further allow him the merit of having done complete justice to the original, as the language, in general, appears to be correct and perspicuous: but we cannot persuade ourselves, that the author has made choice of the best and most advantageous *form* to convey scientific truths truths to the novice in chemiſtry, for whom this elementary work is profeſſedly deſigned. *Aphoriſms*, in our opinion, are not calculated to afford a well-connected or ſyſtematic view of a ſcience, particularly if ſubjective truths are to be demonſtrated by reaſoning upon experiments, for the corroboration of which we avail ourſelves of hypothetical premiſes. Such was formerly the caſe with the *phlogiſtic theory*, and in many points is ſtill ſo with the preſent *antiphlogiſtic ſyſtem*. Both admit a certain *ſubſtratum*; and whether this be in *every caſe demonſtrable*, or not, the theory muſt be ſupported.

In juſtice, however, to Profeſſor Jacquin, it muſt be confeſſed, that his explanations are conciſe, and, in moſt inſtances, ſatisfactory. The work is divided into four principal ſections, although the running number of paragraphs, amounting to 1122, is carried through the whole book; a circumſtance which gives it rather an antiquated appearance. In the firſt ſection, after a ſhort and appoſite *Introduction*, the learned author treats: I. Of chemical ſolution; II. Of chemical affinities; III. Of caloric, or the matter of heat; IV. Of the matter of light; V. Of the atmoſphere; and VI. Of water.

In the next diviſion of the work, the author diſcuſſes "the *mineral kingdom*", the ſubjects of which are carried on from ſection VII. to ſection LXXXIX; or from page 53 to 234.

The third part is devoted to the *vegetable kingdom*", which extends from ſection XC. to CXVI; or from page 235 to 302.

The laſt diviſion contains the "*animal kingdom*", from ſection CXVII. to CXLVI; or from page 309 to 372.—The author then concludes the work with an *Outline of the Phlogiſtic Syſtem*", from page 373 to 381; and an accurate "*Deſcription of Woulfe's Apparatus for Compound Diſtillation*", from page 382 to 405; which is accompanied with a neatly engraved plate, repreſenting the different veſſels uſed in that proceſs.

The tranſlator, laſtly, has provided this intereſting work with a uſeful alphabetical index.

*Opuſcules Chymiques, &c.*—"*Chemical Tracts; being a Sequel to a rational and experimental Syſtem of Chemiſtry*". By BAUME, 8vo. (4 livres.) Paris. Agaſſe.

Theſe 'Tracts' contain the author's obſervations and diſcoveries, made in the courſe of twenty-five years aſſiduous application to this ſcience. Several of theſe papers have already appeared in different periodical publications, but they are now collected in one volume. They contain, in particular, *elucidations reſpecting the cauſe of the cauſticity of quick-lime*, and the *chemical agents* which Macquer attributed to the union of water and earth, and which the author found combined and modified in fire, under an infinite variety of forms. The inquiry into the cauſe of that cauſticity, forms the ſubject of the firſt memoir. The following treat—Of the means of purifying fixed alkalies—Of pot aſh and its purification—Of the fixed ſalt of tartar—Of ſoda—of mineral alkali rendered cauſtic by quick lime—Of the lye of ſoap-wort—Of light.—Several other memoirs, on the conſtruction of thermometers—the diſtillation of ſpirit of wine and mercury—together with other curious and important ſubjects; all which are treated in a manner worthy of the talents and learning of the author, who is well known as one of the moſt diſtinguiſhed chemiſts of the preſent age.

*Chronologiſche Geſchichte der Naturlehre, &c.*—"*Chronological Hiſtory of Natural Philoſophy, continued down to the preſent time.*" Tranſlated from the French of DE LOYS, Member of the Economical Society of Bern; by Dr. C. G.

Dr. C. G. Kuehn, Profeſſor at Leipzig. Vol. I. 310 pp. 8vo. 1798. (1 Rixdollar, or about 3s. 6d.) *Leipzig*. Weygand.

This little work, which is neither ſufficiently accurate nor complete, did not deſerve the honor to be tranſlated by a German Profeſſor. The original appeared at Straſburgh, in 1786; and although the Germans (as well as the Engliſh) poſſeſs no ſatisfactory works on this intereſting ſubject, it cannot be juſtified nor commended, to multiply ſuch haſty compilations and ſuperficial works, by tranſlating and reprinting them in other countries. If Profeſſor Kuhn had ſupplied the deficiencies, and corrected the numerous errors of this 'Chronological Hiſtory' with his uſual ſagacity and induſtry, we ſhould not have heſitated ſtrongly to recommend the tranſlation of ſuch a book into the Engliſh language; but, in its preſent ſtate, we truſt the Engliſh preſs will not be debaſed by an attempt to introduce it to our market.

